# Numerical Study on the Tensile Performance of Headed Stud Shear Connectors with Head-Sectional Damage

**DOI:** 10.3390/ma15082802

**Published:** 2022-04-11

**Authors:** Xiaoqing Xu, Shanwen Zeng, Wei He, Zhujian Hou, Dongyang He, Tao Yang

**Affiliations:** 1 College of Civil Engineering, Tongji University, Shanghai 200092, China; xq.xu@cqu.edu.cn; 2 Guangxi Key Laboratory of Disaster Prevention and Engineering Safety, Nanning 530004, China; 3 Key Laboratory of Disaster Prevention and Structural Safety of Ministry of Education, Nanning 530004, China; 4 Sichuan Xiongzhou Industry Co., Ltd., Chengdu 641400, China; 622170970072@mails.cqjtu.edu.cn; 5 Sichuan College of Architectural Technology, Deyang 618000, China; hewei4912058@163.com; 6 Guangxi Lewang Expressway Co., Ltd., Nanning 530004, China; 622180085002@mails.cqjtu.edu.cn; 7 School of Civil Engineering, Chongqing Jiaotong University, Chongqing 400045, China; 622190082002@mails.cqjtu.edu.cn; 8 College of Civil Engineering and Architecture, Guangxi University, Nanning 530004, China

**Keywords:** headed stud, tension loading, damage, corrosion, concrete cone capacity, simulation

## Abstract

An extensive numerical study was carried out due to the concern that head-sectional damage caused by corrosion poses a threat to the tensile performance of headed stud connectors. Three-dimensional finite element models of pull-out tests were established, with both material and geometric nonlinearities being considered. In particular, the concrete weak region due to bleeding was simulated. The simulation method was verified by the results of pull-out tests on two connectors with different damage degrees. Tensile performance of headed stud shear connectors of various shaft diameters (*d*_s_ = 10 to 25 mm) with various damage degrees (up to 50%) was simulated. It was observed that the connector with a high damage degree exhibited low capacity and a failure closer to pull-out failure than concrete cone breakout failure. Based on the numerical results, reduction factors for quantitatively assessing the influence of head-sectional damage degree on the loading capacity and stiffness of connectors were proposed. With reference to the Concrete Capacity method, the reduction in tensile capacity of connectors with head-sectional damage was found to be caused by the decrease in the projected area of the concrete cone due to the reduction in head diameter, concrete cone angle, and embedment depth. Meanwhile, numerical results showed that the stiffness of a connector at a high embedment depth or in high strength concrete was more sensitive to head-sectional damage. It was also found that the elastic modulus of the weak region significantly affected the stiffness of connectors, while the influence of its thickness on the capacity and stiffness was insignificant.

## 1. Introduction

In steel and concrete composite structures, shear connectors are an essential component for ensuring the composite action. Once the connection between steel and concrete loses its integrity, the integrity of the entire structure comes into jeopardy [1]. Therefore, shear connectors should have sufficient strength and stiffness during the entire design life to enable the steel and concrete components to be designed as parts of a single structural member [2]. Damage to shear connectors caused by static and fatigue load, corrosion, etc. during their service life is inevitable. The damage causes the mechanical properties of the connector to degrade. However, the existing design method is based on the results of tests on the non-damaged connectors, which may result in a reduction of the reliability index if the influence of damage is not considered [3].

**Figure 1:**
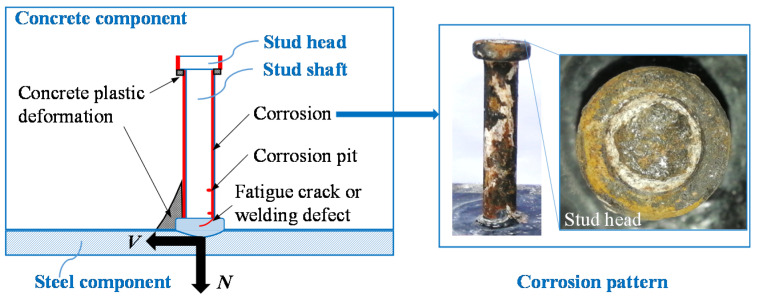
Damage to headed stud shear connectors.

Headed stud shear connectors are the most common type of shear connector [4], and many studies focused on the mechanical properties of damaged headed stud shear connectors have been reported, as shown in Figure 1. The fatigue tests on headed stud shear connectors by Hanswille et al. [5,6] showed that due to the propagation of fatigue crack at stud root, the connector’s residual shear capacity decreases with the increase in number of fatigue cycles. It was reported by Rong et al. [7] that the shear capacity of the connector was not affected by the concentrated corrosion at the stud head, while for headed studs with other corrosion patterns, the detrimental influence of corrosion on static shear capacity and fatigue strength have been reported [8,9,10]. As for welding defects, the study by Han et al. [11] implied that the defects at the bottom of the stud root section is more dangerous with respect to the ultimate slip and ductility.

The above-mentioned studies are all about the shear performance of damaged headed stud shear connectors, and as far as the authors know, there is no research focusing on the adverse effect of damage on the tensile performance of the headed stud shear connector. It has been reported by authors [12] that there would be highly localized corrosion in the stud head when chloride ions penetrate from the exposed surface of the concrete slab to the headed stud. In view of the fact that the stud head is the most key part of the headed stud when resists uplift [13,14], damage to the stud head poses a threat to its tensile performance. Therefore, the influence of head-sectional damage on tensile performance of the connector needs clarification.

In the present study, an extensive numerical study was carried out to quantitatively assess the influence of damage degree on the loading capacity and stiffness of connectors with different sizes. Pull-out tests on single-headed stud shear connectors with head-sectional damage were conducted to verify the simulation method. Based on the numerical results, capacity and stiffness reduction factors were proposed.

## 2. Pull-Out Tests

### 2.1. Specimens

Two-headed stud shear connectors were tested under tension loading, as shown in Table 1. Standard headed studs of 19 mm diameter (*d*_s_) were used. The nominal diameter of the stud head (*d*_h_) was 32 mm. The damage degree of the section at the stud head (*ρ*_h_) was the variable. *ρ*_h_ was calculated by (1-*A*_h,d_/*A*_h,0_), where *A*_h,0_ was the nominal cross-sectional area of the stud head and *A*_h,d_ was the measured real value. NC19-2 specimens adopted the corroded headed studs after the accelerated corrosion tests conducted by authors [12]. For NC19-38 specimen, the headed stud with a greater *ρ*_h_ was obtained by mechanically grinding the stud head.

**Figure 2:**
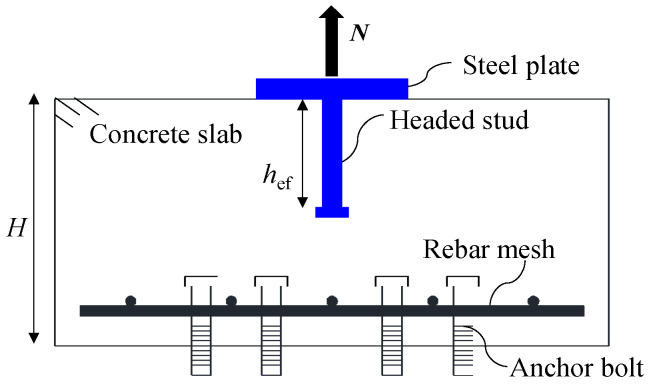
Schematic representation of the specimen.

Figure 2 shows the schematic diagram of the specimen. The headed stud was welded on the center of a steel plate. Embedded anchor bolts of 20 mm diameter were used to anchor the specimen to a I-shaped steel beam during the test. Bottom rebars of 10 mm diameter were used for improving the tension capacities of anchor bolts.

### 2.2. Test Setup

**Figure 3:**
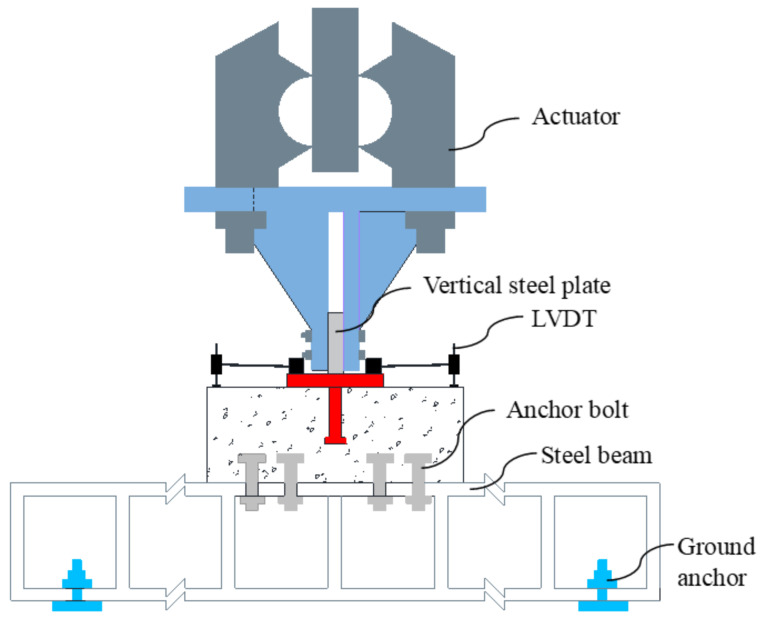
Test setup.

The test setup is shown in Figure 3. The pullout loading was applied to one vertical steel plate welded in the middle of the horizontal steel plate in the specimen. The specimen was connected to a I-shaped steel beam through anchor bolts. The applied load was automatically recorded by the loading machine. For the measurement of the displacement of the stud relative to the concrete surface, four linear variable displacement transformers (LVDTs) with a resolution of 1μm were vertically installed at the corners of the horizontal steel plate symmetrically through magnetic bases.

### 2.3. Material Properties

The standard headed stud [15] was made of ML15 steel [16] with a yielding strength of 450 MPa and an ultimate strength of 495 MPa. The steel plate was made of Q370QD steel with a nominal characteristic yielding strength of 370 MPa. The cube compressive strength and splitting tensile strength of concrete were measured to be 52.49 MPa and 3.85 MPa both through tests on three concrete cubes with sides of 150 mm.

## 3. Experimental Results

**Figure 4:**
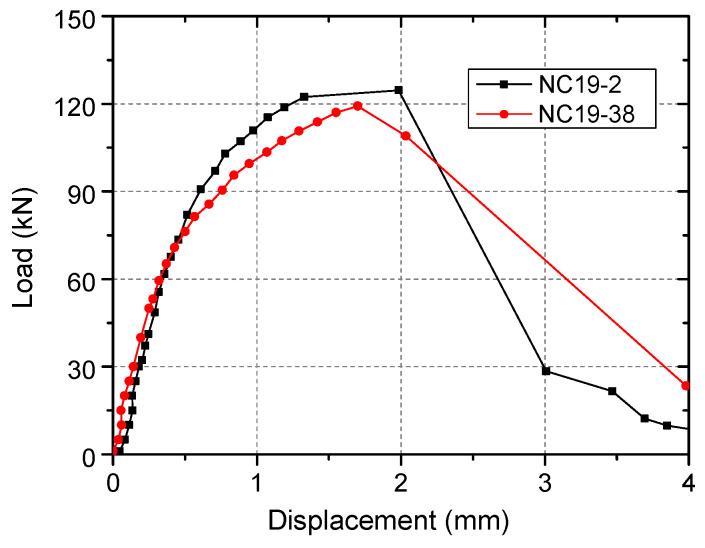
Load–displacement curves of specimens.

The load–displacement curves of specimens are shown in Figure 4. Experimental results including the ultimate load (*N*_u_) and the displacement at the ultimate load (*δ*_u_) are summarized in Table 2. The capacity and stiffness of NC19-38 specimen was smaller, indicating that the excessive head-sectional damage degree reduces the capacity and ductility of headed stud shear connectors in tension, which was consistent with the experimental results by other researchers [13,14].

**Figure 5:**
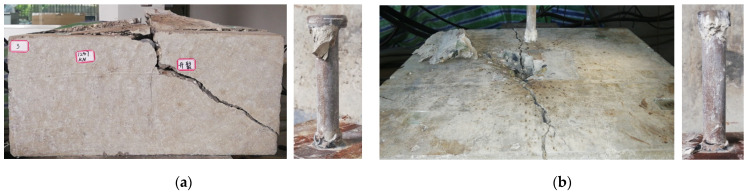
Failure modes of specimens after the studs were pulled out: (**a**) NC19-2; (**b**) NC19-38.

**Figure 6:**
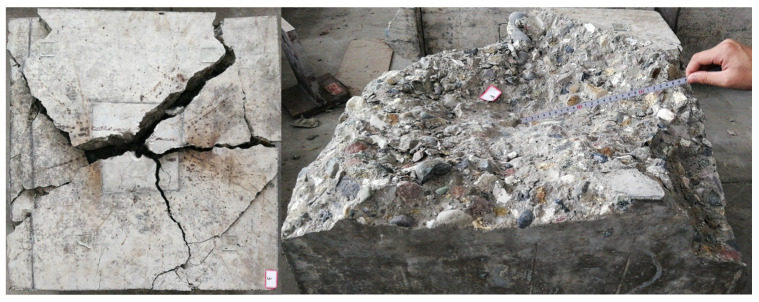
Concrete cone failure mode of NC19-2 specimen.

The failure modes of specimens after the studs were pulled out are shown in Figure 5. NC19-2 specimens failed via concrete cone failure, as shown in Figure 5 and Figure 6. The failure mode of NC19-38 specimen is different. The concrete cone in this specimen was small and the cone surface formed was shallow. Therefore, the failure mode was close to pull-out failure mode.

## 4. Numerical Investigation

### 4.1. Establishment of Finite Element Models

To study more thoroughly the performance of head-damaged stud shear connectors under tensile load, finite element models of pull-out tests were established with a commercial finite element package ABAQUS [17]. In view that the model solving involves complex material nonlinearity, contact, and other nonlinear problems, the dynamic explicit technique in the software was adopted.

#### 4.1.1. General

**Figure 7:**
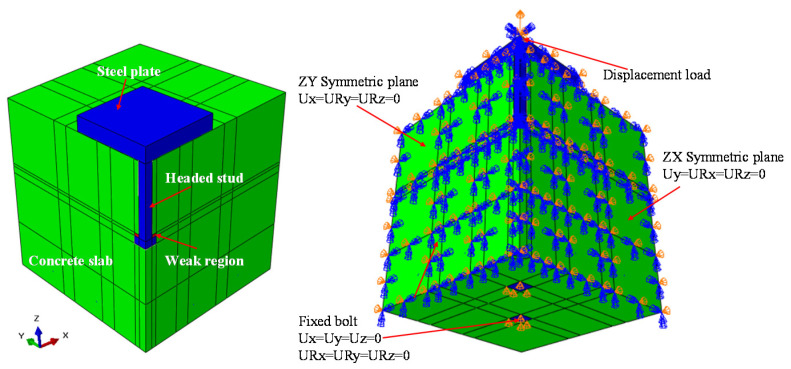
Geometry of finite element model.

Due to the symmetry of the specimen, only a quarter of the specimen was modeled. Figure 7 presents the finite element model where headed stud, concrete slab, steel plate, rebar, and anchor bolts have been simulated. The displacement normal to the plane of symmetry was set to be zero. Vertical displacement load was applied to the top surface of the steel flange, and the anchor bolts were fixed. The surface-to-surface contact was established for all the interfaces. The coefficient of friction was taken as 0.3, which was a commonly used value in simulation of the interface between steel and concrete [18].

#### 4.1.2. Material Modeling

• Concrete

The failure process of the connector under tension loading is greatly affected by the concrete properties. The tensile stiffness degradation of the connector is directly related to the concrete plastic deformation or cracking. When the connector failed in concrete cone breakout, concrete tensile properties are the main factor affecting the loading capacity, while the properties of concrete under local loading are the main factor when the headed stud failed in pulled-out failure mode. Therefore, it is necessary to carefully determine the constitutive model for concrete material.

The plasticity-based constitutive model for the concrete (concrete damaged plasticity model, CDP model) in ABAQUS software was employed to simulates concrete plastic deformation, cracking, and crushing in compression. The yield function, flow rule, hardening rule, and uniaxial stress–strain curves need to be defined for the model.

**Figure 8:**
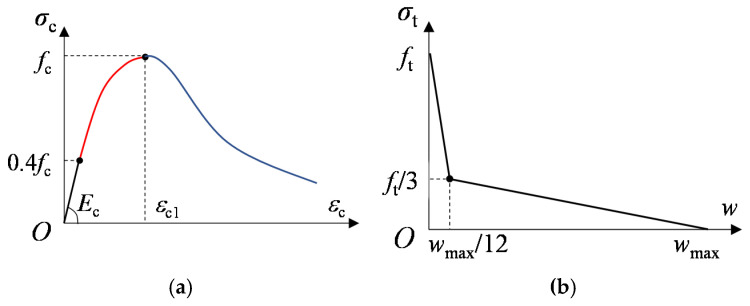
Concrete constitutive model: (**a**) Uniaxial compression; (**b**) Uniaxial tension.

For concrete in compression, a stress–strain curve which consists of three parts was adopted, as shown in Figure 8. In the first part, the compressive stress is assumed to be linear with respect to strain up to the elastic limit stress of 0.4*f*_c_. Beyond this point, concrete plastic deformation occurs. The second part of curve is nonlinear and can be determined by Equation (1). The third part of the curve is a nonlinear descending part, and the compressive stress is represented as a function of strain according to Equation (2), as suggested by [19].

(1) $$\sigma_{c}=\left(\frac{kn-n^{2}}{1+\left(k-2\right)n}\right)f_{c}$$

(2) $$\sigma_{c}={\left(\frac{2+\gamma_{c}f_{c}\varepsilon_{c1}}{2f_{c}}-\gamma_{c}\varepsilon_{c}+\frac{\gamma_{c}\varepsilon_{c}^{2}}{2\varepsilon_{c1}}\right)}^{-1}$$

where, $k=E_{c}\varepsilon_{c}/f_{c}$; $n=\varepsilon_{c}/\varepsilon_{c1}$; $\varepsilon_{c1}$ is the strain at maximum compressive stress; $\gamma_{c}$ is the only free parameter controlling the area under the stress–strain curve and it was taken to be 1.7 [20].

For concrete in tension, the tensile stress is assumed to be linearly with respect to strain up to the concrete tensile strength. Beyond this point, concrete crack initiates. Then, a bi-linear tensile stress–crack width diagram is assumed, as shown in Figure 8. Here, the maximum crack width (*w*_max_) was taken as 0.18 mm [21].

Besides uniaxial compressive and tensile constitutive model, there are several key parameters in CDP model determining the yield function, flow rule, and hardening rule [17]. By reference to existing research on numerical modeling of headed stud shear connectors [18,22], values of these concrete material parameters used in this study are listed in Table 3.

• Headed stud, structural steel, and reinforcing steel

**Figure 9:**
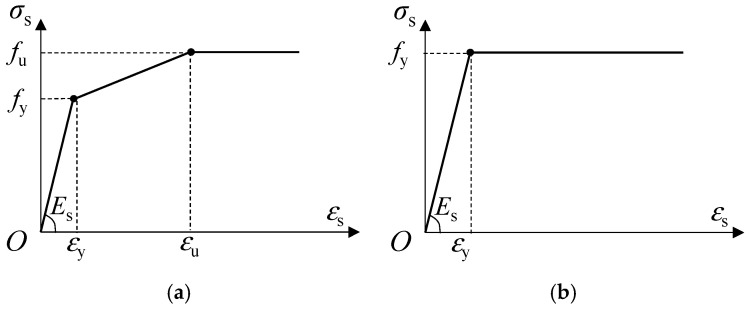
Steel material model for: (**a**) Headed stud; (**b**) Structural steel and reinforcing steel.

A tri-linear model was used to model the steel material of headed stud. The values of elastic modulus (*E*_s_), yield strength (*f*_y_), and ultimate strength (*f*_u_) were the same as the test values given above, and the ultimate strain (*ε*_u_) was taken as 0.6%, respectively. The stress–strain relationship for steel is presented in Figure 9. For simplicity, a bilinear stress–strain model was used to model the stress–strain relationship of structural steel and reinforcing steel, as shown in Figure 9. The parameters of their mechanical properties mentioned above were used. For all the steel material, it is assumed that the mechanical behavior for both compression and tension is the same.

#### 4.1.3. Modeling the Concrete Weak Region Due to Bleeding

**Figure 10:**
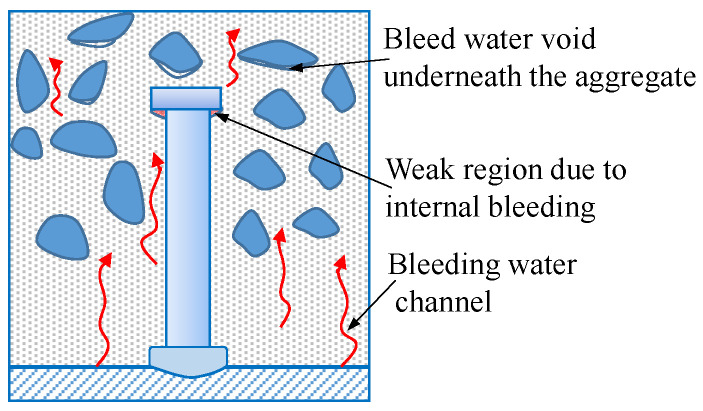
Weak region due to internal bleeding in the concrete slab.

During the concrete pouring in this study, the headed stud was placed with its head on the top, opposite to the direction in which the headed stud was placed during the loading test. This concrete pouring method imitated the method adopted in the actual construction of steel and concrete composite girders. The stud head blocked the movement of the free water to the upper direction of the freshly placed concrete, as shown in Figure 10. As a result, bleeding occurred underneath the stud head which adversely affected the concrete there by increasing the porosity, hence reducing the strength and elastic modulus [23]. The worse consequence is that there may be a gap between the stud head and the concrete.

As shown in Figure 7, in order to simulate the weak region (WR) with low stiffness underneath the stud head, this region in the model was assigned an elastic material with a lower modulus of elasticity than concrete. The elastic material does not yield, which can approximately simulate the increase in the yield strength of this region after being compacted under tri-axial compression. The reason for not adopting a more complex CDP model is to avoid the difficulty of convergence. At the same time, no studies on effectively simulating this weak region have been reported, and it is questionable whether the CDP model is applicable.

**Figure 11:**
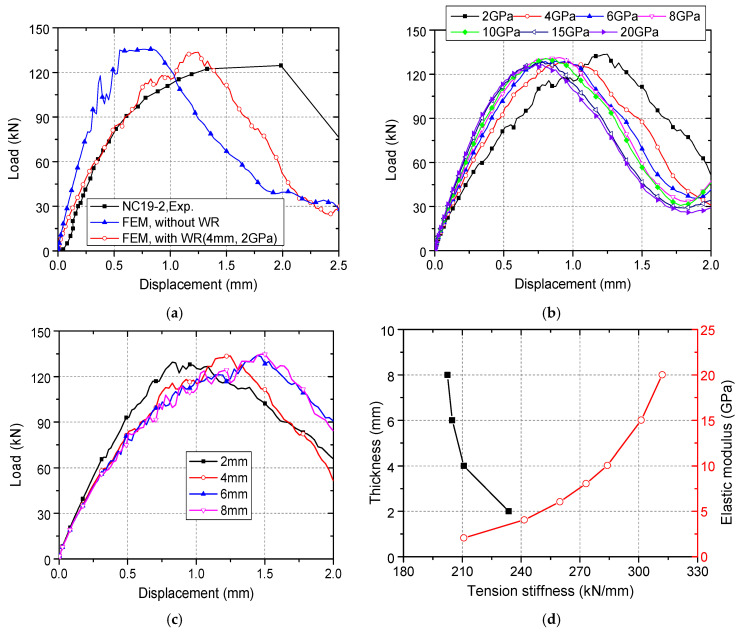
Effect of WR on the calculated results: (**a**) Calculated load–displacement curves of models with or without WR; (**b**) Results of models with different WR elastic moduli; (**c**) Results of models with different WR elastic thicknesses; (**d**) Effect of WR on tensile stiffness of connectors.

Figure 11a shows the comparison between simulation results of models with or without weak region with the experimental results. Here, the thickness of the weak region with elastic modulus of 2 GPa is 4 mm. The difference in ultimate load between the models is small, because WR does not change the shape of the concrete cone. The deformation performance of the connector has changed significantly. Comparing the secant stiffness at the displacement of 0.2 mm, it can be seen that after simulating the WR, the calculated stiffness of the connector is reduced by 57.3%, which is closer to the stiffness obtained by the test. This indicates that the concrete slab cannot be regarded as homogeneous, and it is necessary to consider the decrease in the stiffness of the connector caused by WR. Therefore, in the subsequent models, WR was simulated.

Figure 11b,c respectively compare the load–displacement curves of models with different WR elastic moduli (while the thickness is unified to 4 mm) and thicknesses (while the elastic modulus is unified to 2 GPa). The influence of WR elastic modulus and thickness on the ultimate load can be ignored. As shown in Figure 11d, the tensile stiffness of the connector increases as the WR elastic modulus increases; the tensile stiffness increases as the WR thickness decreases. Based on the comparison between the simulation results with the test results, as shown in Figure 11a, the WK thickness is taken as 4 mm and the elastic modulus is taken as 2 GPa finally.

#### 4.1.4. Mesh

For concrete slab, 10-node modified quadratic tetrahedron elements (C3D10M) were used in order to achieve an accurate result from these analyses with large amounts of plastic deformation. Moreover, C3D10M was used for modeling anchor bolts in order that they shared nodes with the concrete slabs in the interface, because the slip between anchor bolts and concrete slab were not paid attention to. For the headed stud and steel plate, three dimensional eight-node elements (C3D8R) with a linear approximation of displacements, reduced integration with hourglass control eight nodes, and three translational degrees of freedom were adopted to improve the rate of convergence. For rebars, two-node linear three-dimensional truss elements (T3D2) with linear approximation of displacement, two nodes, and three translational degrees of freedom were used.

### 4.2. Validation Studies

**Figure 12:**
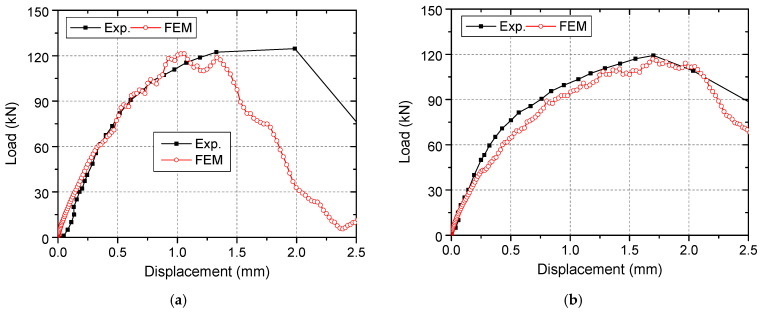
Comparison between calculated and experimental load–displacement curves: (**a**) NC19-2; (**b**) NC19-38.

The predicted numerical responses of specimens are compared with test results in Figure 12. In terms of the initial stiffness and the ultimate load, the simulation results show good agreement with the test ones. It can be observed that head-sectional damage had a negative influence on the tensile behavior of the specimens, which has been found in the tests. However, the displacement at the ultimate load was underestimated by the numerical simulations, which is attributed to the inherent idealizations of the CDP model in modelling the concrete material.

**Figure 13:**
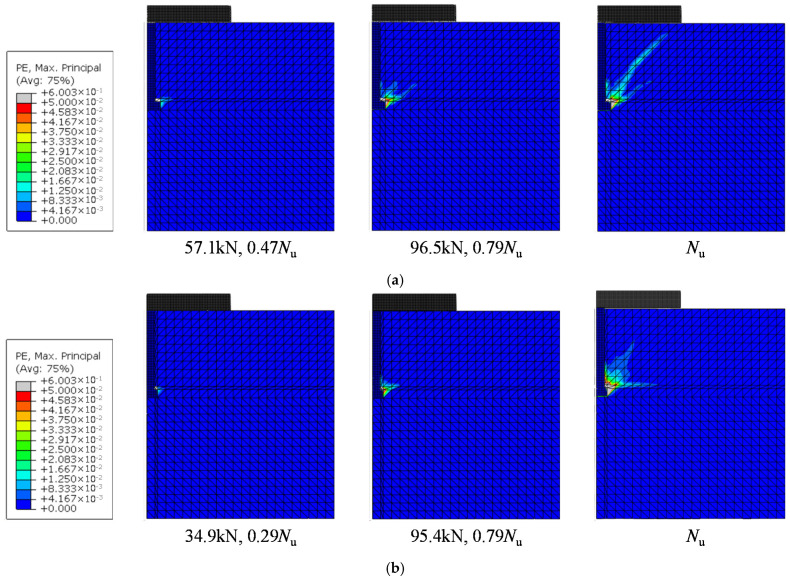
Maximum principal plastic strain at different loading stages: (**a**) NC19-2; (**b**) NC19-38.

One of the important differences between the test specimens is the failure mode. For further validation, attention was paid to the failure mode predicted by the numerical simulations. As shown in Figure 13, two typical failure modes occurred in the tests were well predicted by the numerical models. The model for NC19-2 specimen predicted the circumferential crack around the stud head. In the model for NC19-38 specimen, concrete cone did not form at the ultimate load and large plastic strain was observed above the stud head, indicating a pull-out failure.

The above-described validation procedure demonstrates the reliability of the employed modelling procedure and corresponding key parameters selected for the concrete damage plasticity model in this study.

## 5. Parametrical Analysis and Proposed Reduction Factors

The number of specimens in the experimental study was limited. Therefore, in order to further investigate the influence of head-sectional damage on the tensile performance of the connector, a number of finite element models of connectors with different shaft diameters and head diameters (i.e., different head-sectional damage degrees) were established. The remaining main parameters of these models were consistent with the models for tested specimens mentioned above.

**Figure 14:**
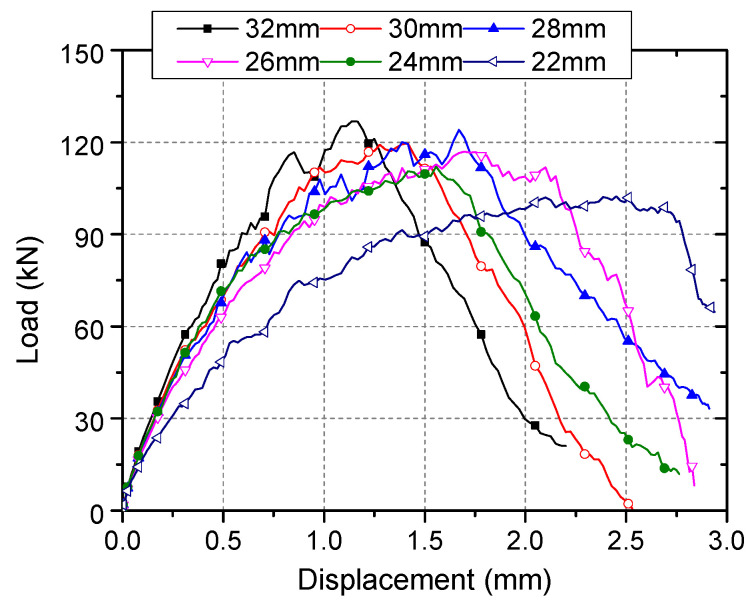
Results of models with different sizes of stud head.

Figure 14 shows the calculated load–displacement curves of some models of connectors with 19 mm shaft diameter as an example. Based on these calculated deformation results as well as the results of stress and strain, the influence of damage degree on the failure mechanism, tensile capacity, and stiffness of connectors was analyzed, and the calculation method of tensile capacity and stiffness of damaged connectors was proposed.

### 5.1. Failure Mechanism

The size of the stud head affects the stress and strain state of the model. As a result, concrete cracking process and the formation and development of concrete cone is affected, and ultimately the failure mode of the connector could be changed.

**Figure 15:**
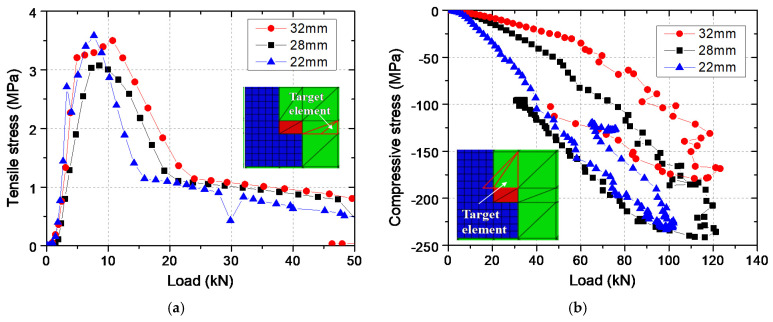
Stress of critical concrete elements: (**a**) Tensile stress; (**b**) Compressive stress.

The concrete near the stud head cracked at the beginning of loading, and the smaller the head diameter, the cracking was earlier and faster. Figure 15a shows the calculation results of the tensile stress of the concrete element closest to the stud head. Obviously, in the model with a stud head diameter of 22 mm, the tensile stress increased the fastest with the load and the cracking load was the smallest. However, cracking loads of models with different stud head diameters were all about 9 kN, and the maximum difference between the specimens was only about 3 kN. The cracking load was less than 10% of the tensile capacity, and after the load continued to increase by 10 kN. The crack width reached about 0.015 mm according to the concrete tensile stress–crack width diagram in Figure 15. Eligehausen and Sawade [24] believed that tension crack forms at about 30% of the ultimate load based on the test results of concrete strain in the concrete slab. However, in their tests, the concrete strain gauges were not arranged in the immediate vicinity of the stud head, which made the initial cracking load to be overestimated. When the head diameter is smaller, the bearing stress of the concrete above the stud head increases faster with the load. Figure 15b shows the compressive stress of the concrete element directly above the stud head. The compressive stress in the model with a head diameter of 22 mm had the fastest increase, and the compressive stress at the load of 100 kN was 1.39 and 2.3 times that of the models with 28 mm and 32 mm head diameters, respectively. However, the maximum compressive stress of this model at the time of failure was 230 MPa, which was smaller than that of the model with 28 mm head diameter.

### 5.2. Tensile Capacity

**Figure 16:**
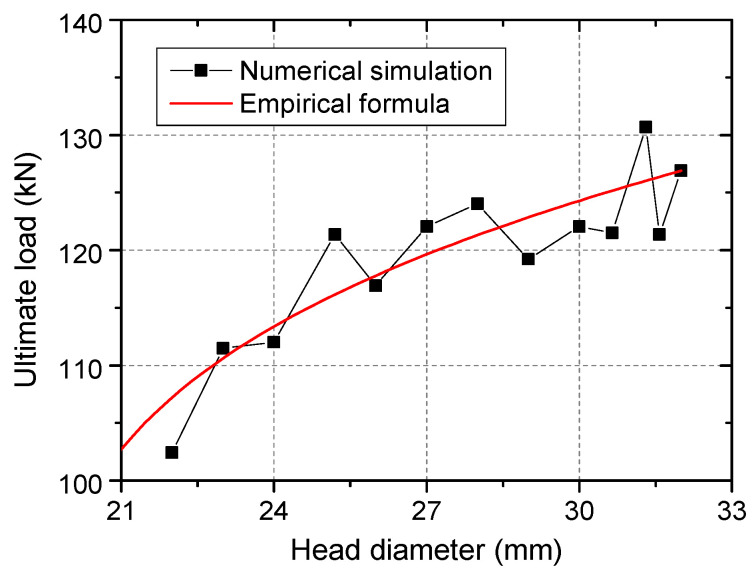
Comparison between calculation results of numerical simulation and empirical formula.

Calculated tensile capacities of models with different stud head sizes are presented in Figure 16. It is obvious that the tensile capacity decreases with the decrease of the stud head diameter. In order to assess the influence of head-sectional damage on the tensile capacity quantitatively, an empirical reduction factor for evaluating the tensile capacity of the damaged shear connector was proposed, and the mechanism of performance degradation was analyzed by Concrete Capacity (CC) method [25].

• Proposed capacity reduction factor

The direct cause of the reduction in the tensile capacity is the decrease of the bearing area. Thus, the capacity reduction factor can be written as a function of the bearing area. Nilforoush et al. [13] have proposed a modification factor to consider the anchor-head size on the tensile capacity of headed anchors in uncracked concrete as follows:

(3) $$N_{c}=\psi_{\mathrm{AH}}N_{c,m}$$

(4) $$\psi_{\mathrm{AH}}={\left(\frac{A_{b}}{A_{b}^{\mathrm{code}}}\right)}^{0.1}$$

where *N*_c,m_ is the standard concrete cone breakout capacity; *N*_c_ is the modified concrete cone breakout capacity; *A*_b_ is the anchor bearing area; *A*_b_^code^ is the equivalent bearing area corresponding to a bearing stress of *σ*_b_ = 15*f*_c_ under the anchor head at peak load, which can be determined from Equation (5) [13].

(5) $$A_{b}^{\mathrm{code}}=\frac{16.8\sqrt{f_{c}}h_{\mathrm{ef}}^{1.5}}{15\cdot f_{c}}$$

where *f*_c_ is the concrete compressive strength.

Then, the ratio of tensile capacities of connectors after damage and before damage is obtained.

(6) $$\eta_{d,\mathrm{str}}=\frac{N_{c,d}}{N_{c,0}}={\left(\frac{A_{b,d}}{A_{b,0}}\right)}^{0.1}$$

where *A*_b,0_ is the original bearing area; *A*_b,d_ is the bearing area after damage and is the function of the damage degree:

(7) $$A_{b,d}=A_{h,d}-A_{s,0}=A_{h,0}\left(1-\rho_{h}\right)-A_{s,0}$$

where *A*_h,0_ and *A*_h,d_ are the cross sectional area of stud head before and after damage; *A*_s,0_ is the cross sectional area of stud shaft. Thus, the capacity reduction factor is written as follows.

(8) $$\eta_{d,\mathrm{str}}={\left[\frac{A_{h,0}\left(1-\rho_{h}\right)-A_{s,0}}{A_{h,0}-A_{s,0}}\right]}^{0.1}$$

Tensile capacities of damaged connectors were calculated according to Equation (8) and were drawn with a red line in Figure 16. The influence of head-sectional damage on the tensile capacity was well predicted. The calculated capacity to simulated capacity ratio has a mean value of 1.004 and a standard deviation of 0.030.

**Figure 17:**
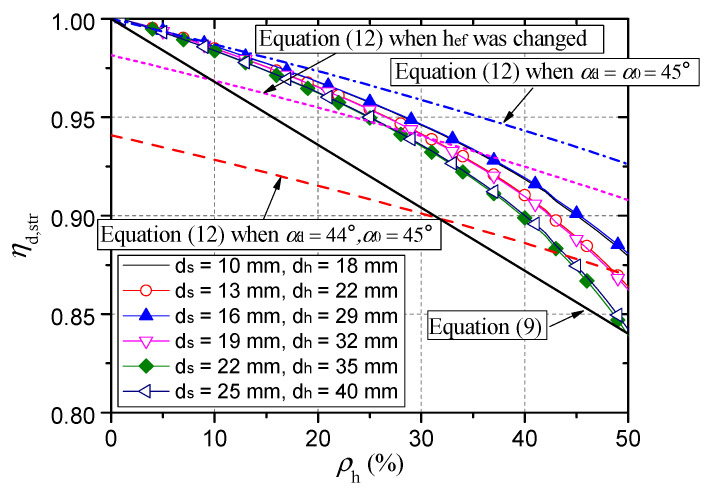
Calculated capacity reduction factors.

The key dimensions such as the head diameter and the shaft diameter of the standard headed studs of different specifications have been specified in standards [15]. In order to facilitate the design of engineers, a simple formula applicable to all types of standard headed studs is required. It is worth noting that for standard headed studs, a corrosion degree greater than 50% results in the head diameter being close to the shank diameter. In practice, such severe corrosion should be avoided. As a result, the upper limit of the corrosion degree discussed here is 50%. Figure 17 shows calculated capacity reduction factors when the corrosion degree was less than 50%. For headed studs with shaft diameters of 22 mm and 25 mm, the reduction factor decreases the fastest, indicating that their tensile capacities are the most sensitive to head-sectional damage. For all types of headed studs, the reduction factor and the damage degree basically show a linear relationship when the degree is less than 30%, and when the degree reaches 50% the reduction factor is generally smaller than 0.84. In order to calculate the reduction factor easily and in the safe side, Equation (9) was suggested. In Figure 17, the formula is presented with a black straight line, one of whose endpoints is (50%, 0.84).

(9) $$\eta_{d,\mathrm{str}.}=1-0.32\rho_{h}$$

• Theoretical analysis on reasons for the capacity reduction

**Figure 18:**
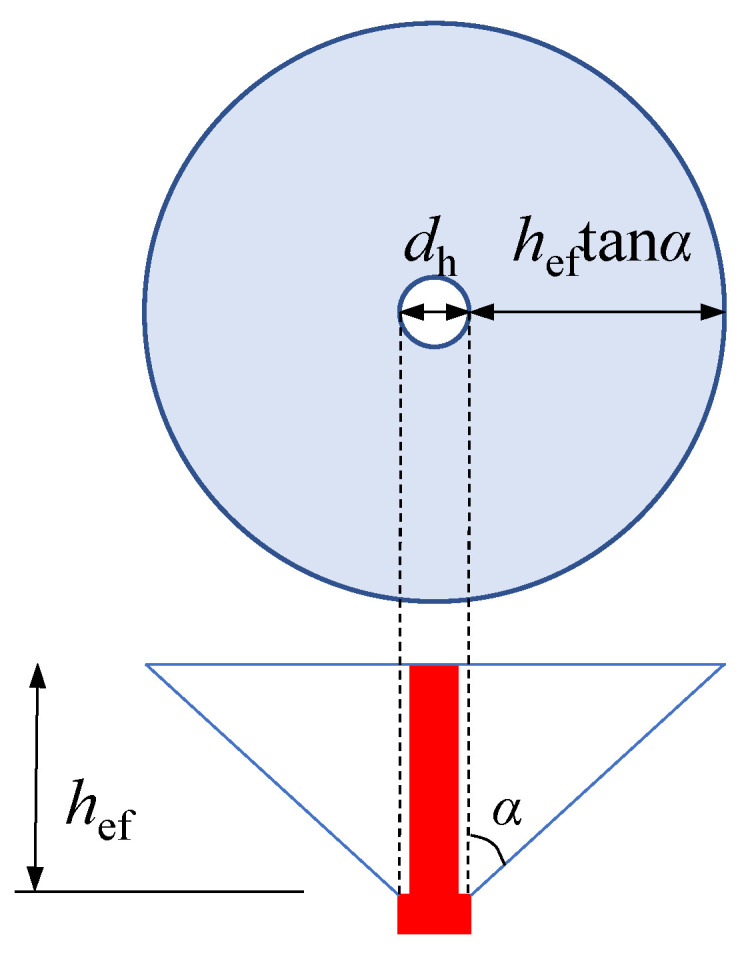
Idealized concrete cone and its projected area.

According to the CC method, the concrete cone breakout capacity is proportional to the projected area of the concrete cone, as shown in Equation (10).

(10) $$N_{c}=A_{c}f_{t}$$

(11) $$A_{c}=\pi {\left(h_{\mathrm{ef}}\tan\alpha +\frac{d_{h}}{2}\right)}^{2}-\frac{\pi d_{h}^{2}}{4}$$

where, *A*_c_, as shown in Figure 18, is the projected area; *α* is the semi vertical angle of the cone.

The head-sectional damage affects the shape of the concrete cone and reduces its projected area. When the damage degree is *ρ*_h_, the diameter of the stud head is $\left(\sqrt{1-\rho_{h}}\cdot d_{h}\right)$, and the reduction factor calculated according to CC method is as follows.

(12) $$\eta_{d,\mathrm{str}.}=\frac{h_{\mathrm{ef},d}^{2}\tan^{2}\alpha_{d}+\sqrt{1-\rho_{h}}d_{h}h_{\mathrm{ef},d}\tan\alpha_{d}}{h_{\mathrm{ef},0}^{2}\tan^{2}\alpha_{0}+d_{h}h_{\mathrm{ef},0}\tan\alpha_{0}}$$

where *h*_ef,0_ and *h*_ef,d_ are the effective embedded depths before and after damage, respectively; *α*_0_ and *α*_d_ are the angles before and after damage, respectively.

The reduction in head diameter is not the only reason for the reduction in tensile capacity. The blue dash-dot line in Figure 17 shows the calculated results of Equation (12) when both *α*_d_ and *α*_0_ are 45°. When the damage degree is less than 20%, this curve is close to the curve calculated by the empirical formula, Equation (8), while the tensile capacity is greatly overestimated when the damage degree is greater than 20%. This indicates that there are other reasons for the reduction in tensile capacity.

Another important reason is that the concrete cone becomes steeper. The foregoing simulation results in this paper, as well as the experimental and simulation results reported in the literature [13,14], show that as the head size decreases, *α* of the concrete cone decreases slightly, that is, *α*_d_ in Equation (12) is also a function of *ρ*_h_. The red dashed line in Figure 17 illustrates the calculated results of Equation (12) when *α*_d_ and *α*_0_ are 44° and 45°, respectively. The curve is generally below all the curves calculated by Equation (8), thereby confirming the significant influence of *α* on the tensile capacity.

Smaller embedment depth at failure is another key reason for the reduction in tensile capacity. The displacement corresponding to the ultimate load of the damaged connector increases with the increase of the damage degree, as shown in Figure 14, indicating that the local concrete crushing is more serious for a connector with a larger degree of head-sectional damage. As a result, *h*_ef,d_ is less than *h*_ef,0_ [14]. The purple dotted line in Figure 17 represents the calculated results of Equation (12) when *h*_ef,d_ and *h*_ef,0_ takes 94 mm and 95 mm, respectively, and both *α*_d_ and *α*_0_ are 45°. It can be seen that the reduction factor is further reduced.

### 5.3. Tensile Stiffness

The tensile stiffness of a connector is a key parameter to be used in the design of steel and concrete composite structures. The tensile stiffness of each connector in grouped connectors determines the tensile force distribution between the connectors. Meanwhile, it affects the overall rigidity of composite structures.

The foregoing research has pointed out that the tensile stiffness decreases with the increase in the degree of head-sectional damage. However, the adverse effect of head-sectional damage on tensile stiffness has not been paid attention to by Yang et al. [26] who believed that the diameter of the stud shaft and embedment depth were the main factors that influenced the tensile stiffness. Therefore, the relationship between the damage degree and the tensile stiffness was investigated.

The measured displacement of the headed stud under tension loading is the sum of the displacement of the head relative to the concrete slab (*δ*_Con_, i.e., the displacement of the head caused by the compression deformation of the concrete) and the tensile deformation of the stud shaft (*δ*_Ste_).

(13) $$\delta =\delta_{\mathrm{Con}}+\delta_{\mathrm{Ste}}$$

Here, corrosion of the stud shaft was not considered. Therefore, only *δ*_Con_ was paid attention to, and the secant stiffness when this displacement was 0.1 mm was defined as the initial stiffness, as shown in Equation (14).

(14) $$k_{t,\mathrm{Con}}=\frac{N_{0.1}}{0.1}$$

where *N*_0.1_ is the load when the calculated *δ*_Con_ is 0.1 mm.

**Figure 19:**
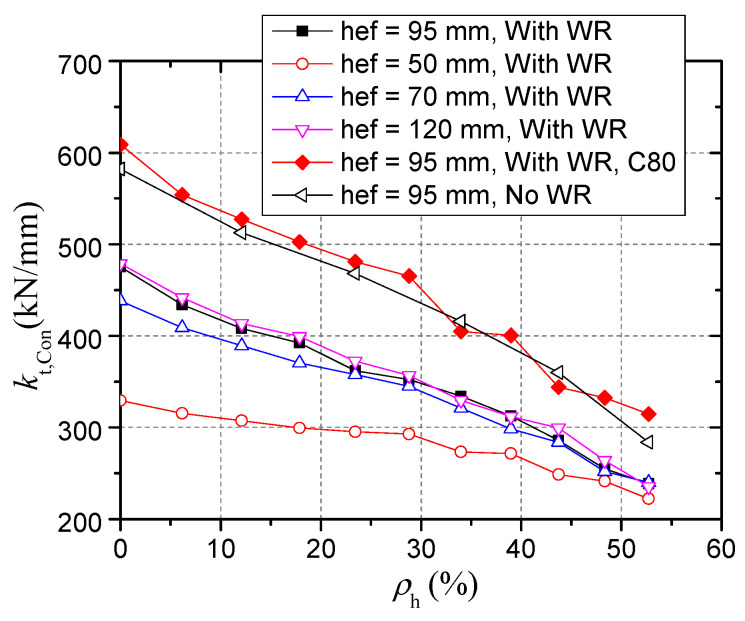
Relationship between the tensile stiffness and the damage degree.

The relationship between the tensile stiffness and the damage degree is basically linear. The black line with black rectangles symbols in Figure 19 shows the tensile stiffness of connectors with embedment depth of 95 mm after head-sectional damage of different degrees.

In order to obtain a widely applicable formula for calculating the tensile stiffness of a damaged connector, the influence of the embedment depth, concrete strength, and the concrete weak region due to bleeding on the tensile stiffness was analyzed through 110 models.

• Effect of embedment depth

For a connector with larger embedment depth, the influence of head-sectional damage on stiffness is larger. Figure 19 compares the results of connectors with embedment depths of 50 mm, 70 mm, 95 mm, and 120 mm. When the embedment depth is 50 mm, the inclination of the calculated curve is the smallest, indicating that the tensile stiffness change caused by the head-sectional damage is the smallest.

This can be explained by analyzing the composition of concrete compression deformation. The strain state of concrete in low-load stage shown in Figure 13 demonstrates that only a small part of concrete surrounding the stud head has plastic deformation due to cracking and high compressive stress, while other concrete is still in elastic. Compression deformation is the sum of elastic deformation (*δ*_Con,E_) and plastic deformation (*δ*_Con,P_) of concrete, as shown in Equation (15). The concrete plastic deformation would increase due to the increase in bearing stress when the damage degree increases. Since the proportion of concrete plastic deformation in the total concrete deformation of the connectors with smaller embedment depths is greater, under the same bearing stress increase, the increase of plastic deformation in these connectors is relatively slow. As a result, the influence of head-sectional damage on the stiffness is smaller for the connectors with smaller embedment depth. This suggests that the calculation results of the connectors with larger embedment depths can be used to safely evaluate the adverse effects of corrosion on tensile stiffness.

(15) $$\delta_{\mathrm{Con}}=\delta_{\mathrm{Con},E}+\delta_{\mathrm{Con},P}$$

**Figure 20:**
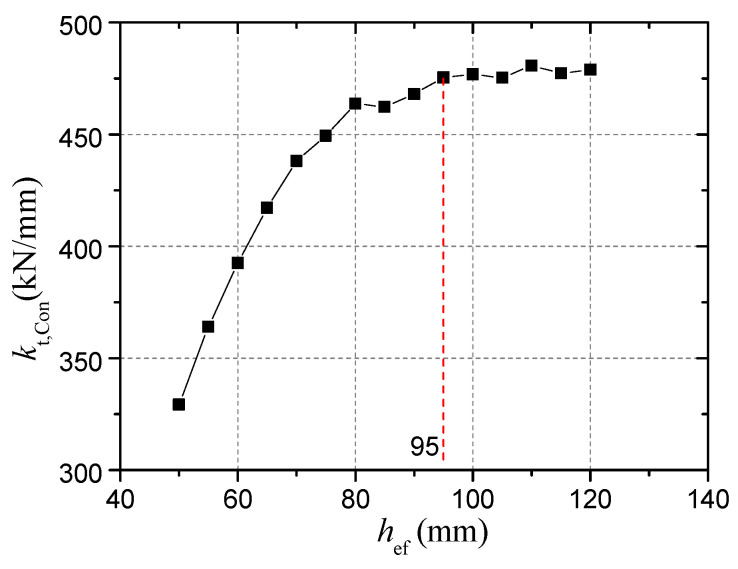
Effect of the effective embedment depth on the stiffness.

Figure 19 shows the variation of tensile stiffness with embedment depth when there is no head-sectional damage in the connectors. When the embedment depth is greater than 95 mm, the stiffness tends to be a constant value. This is inconsistent with the results by Yang et al. [26] who thought that the tensile stiffness always has a linear relationship with *h*_ef_. Furthermore, as shown in Figure 20, for different damage degrees, the stiffness almost does not increase when the embedment depth is large enough.

• Effect of concrete strength

For a connector embedded in high strength concrete, the tensile stiffness is larger and the influence of head-sectional damage on stiffness is larger. Figure 20 shows that when the connector embedded in C80 concrete, the inclination angle of the calculated curve is the largest. Although plastic deformation accounts for a smaller proportion of the total deformation of the connector in C80 concrete, the plastic deformation increases more quickly with the same increase in the bearing stress. Therefore, the calculation results of the damage degree–stiffness relationship when the concrete strength is high can be used to safely evaluate the adverse effect of head-sectional damage on the stiffness of the connector in normal strength concrete.

• Effect of the concrete weak region due to bleeding

**Figure 21:**
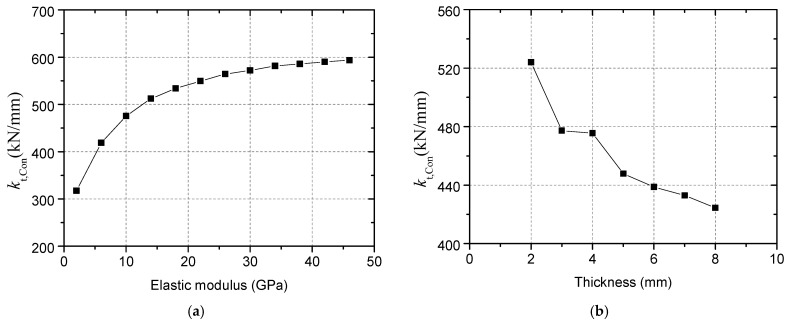
Effect of properties of the weak region on the stiffness: (**a**) Elastic modulus; (**b**) Thickness.

Figure 20 shows the calculation results of models without a concrete weak region. The weak region due to bleeding reduces the tensile stiffness and the influence of head-sectional damage on stiffness. As shown in Figure 21, the stiffness of a connector when the elastic modulus of the region is 46GPa is twice that when the elastic modulus is 2GPa, while the influence of the thickness on the stiffness is insignificant. These calculation results suggest that the experimental results will be significantly affected by the randomness of the properties of the weak region, thus concealing the effect of damage degree on the stiffness.

• Proposed stiffness reduction factor

**Figure 22:**
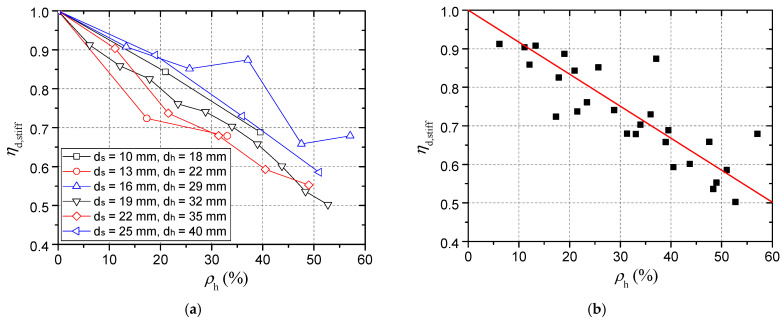
Stiffness results of damaged connectors of different sizes and different damage degrees: (**a**) Stiffness reduction factor; (**b**) Proposed formula of stiffness reduction factor.

Figure 22a shows stiffnesses of damaged connectors of different sizes and different damage degrees. The vertical coordinate in this figure is the ratio of stiffness before and after corrosion, i.e., the stiffness reduction factor. Based on these calculation results, Equation (16) to calculate the reduction factor was obtained by linear regression, as shown in Figure 22b. The calculation model gave a fine fitness with a R-square of 0.695. It is worth noting that this stiffness reduction factor is only for the stiffness of concrete deformation not for the total stiffness. Compared with the capacity reduction factor, it is found that the stiffness reduction factor decreases faster with the increase of the damage degree.

(16) $$\eta_{d,\mathrm{stiff}}=1-0.83\rho_{h}$$

## 6. Conclusions

An extensive numerical study and two pull-out tests were conducted to evaluate the effect of head-sectional damage on the behavior of headed stud shear connectors under tension loading, and the following conclusions were obtained:

(1) Concrete weak region due to bleeding should be considered in the finite element models, and it was found that its elastic modulus significantly affected the stiffness of connectors, while the influence of its thickness on the capacity and stiffness was minor. After modeling a weak region with an elastic modulus of 2 GPa and a thickness of 4mm, the simulation results demonstrated good agreement with the test results in terms of the initial stiffness and the ultimate load.
(2) When the head-sectional damage degree was 38%, the failure mode changed from concrete cone breakout to pull-out failure, resulting in a decrease in loading capacity and ductility. The numerical results show that the load, when concrete, near the stud head cracked decreased with the damage degree, while the crack width increased with the damage degree. The cracking load was found to be less than 10% of the connector’s tensile capacity, which was much smaller than the 30% reported by Eligehausen and Sawade [24]. Meanwhile, when the head diameter reduced from 32 mm to 22 mm, the bearing stress of the concrete above the stud head and shear stress of concrete in the vicinity of the stud head had more than doubled. These differences in stress state and failure process were causes for the change from concrete cone breakout failure to pull-out failure when the damage degree increased.
(3) An equation of capacity reduction factor with the bearing area and damage degree as parameters (Equation (8)) was proposed and verified by numerical results. Furthermore, a concise formula, Equation (9), was suggested to calculate the reduction factor of different types of headed stud shear connectors easily and on the safe side. With reference to the Concrete Capacity method, the decrease in head diameter, concrete cone angle, and embedment depth was found to be the reason for the reduction in tensile capacity of connectors with head-sectional damage.
(4) Tensile stiffness was observed to decrease with the decrease in the stud’s head size, which has not been considered by Yang et al. [26]. A linear relationship between the stiffness reduction factor and head-sectional damage degree was found, and a formula for the stiffness reduction factor (Equation (16)) was obtained by linear regression on the numerical results. The numerical results also showed that the stiffness of a connector at a high embedment depth or in high strength concrete was more sensitive to head-sectional damage. Compared with the capacity reduction factor, the stiffness reduction factor decreased faster with an increase in the damage degree.

## Figures and Tables

**Table 1:** Summary of specimens.

Labels	*d*_s_ (mm)	*d*_h_ (mm)	*h*_ef_ (mm)	*A*_h,d_ (mm^2^)	*ρ*_h_	Concrete Slab Geometry (mm × mm × mm)
NC19-2	19	32	95	783.66	2.6%	550 × 550 × 250
NC19-38	19	32	95	498.76	38.0%	550 × 550 × 250

**Table 2:** Experimental results.

Labels	*N*_u_ (kN)	*δ*_u_ (mm)	Failure Mode
NC19-2	124.7	1.99	Concrete cone failure
NC19-38	119.2	1.70	Pull-out failure

**Table 3:** The employed material parameters for the finite element models.

Poisson’s Ratio	Dilation Angle	Eccentricity Factor	Biaxial-to-Uniaxial Strength Ratio	Shape Factor	Viscosity Parameter
0.16	32	0.1	1.16	0.667	0

## Data Availability

The data presented in this study are available on request from the corresponding author.
